# Preclinical assessment of dual CYP26[A1/B1] inhibitor, DX308, as an improved treatment for keratinization disorders

**DOI:** 10.1002/ski2.22

**Published:** 2021-03-26

**Authors:** J.G.S. Veit, Y. Poumay, D. Mendes, J. Kreitinger, L. Walker, A. Paquet, C. Menigot, F. Zolezzi, A.S. Paller, P. Diaz

**Affiliations:** ^1^ Department of Biomedical and Pharmaceutical Sciences University of Montana Missoula Montana USA; ^2^ URPHYM‐NARILIS University of Namur Namur Belgium; ^3^ R&D DermaXon LLC Missoula Montana USA; ^4^ Syneos Health Biot France; ^5^ Department of Dermatology Northwestern University Feinberg School of Medicine Chicago Illinois USA

## Abstract

**Background:**

Retinoid‐based therapies are commonly used in the treatment of disorders of keratinization and other skin disorders but can result in non‐specific effects and adverse reactions. Use of retinoic acid metabolism blocking agents (RAMBAs) such as DX308 may address these shortcomings.

**Objectives:**

Characterize the therapeutic potential of recently discovered, CYP26‐selective RAMBA, DX308.

**Materials and Methods:**

Preliminary in vitro assessment of potential off‐target activity, metabolic and toxicologic profiling. Studies to assess safety and efficacy of topical treatment in correcting abnormal skin morphology in rhino mice. Extensive gene expression profiling by RNA sequencing and qPCR in 3D epidermis grown with keratinocytes (KCs) from keratinization disorders and healthy controls, to investigate modulation of retinoid biopathways.

**Results:**

In vitro, DX308 does not interact with off‐target nuclear receptors or CYP450s, is not genotoxic, and is stable in skin, despite vigorous hepatic metabolism. In vivo, topical DX308 induces comedolysis and epidermal thickening without apparent adverse effects. Gene expression profiling shows potent modulation of retinoid‐responsive genes by DX308 in both healthy and keratinization disorder KCs. Pathway analysis suggests DX308 may inhibit inflammatory and immune responses in KCs.

**Conclusions:**

These preliminary studies suggest that DX308 is an efficacious topical therapeutic with a favourable metabolic and safety profiles. DX308 may present an improved therapeutic alternative for the treatment of keratinization disorders and other retinoid‐responsive skin ailments.

## Graphical abstract

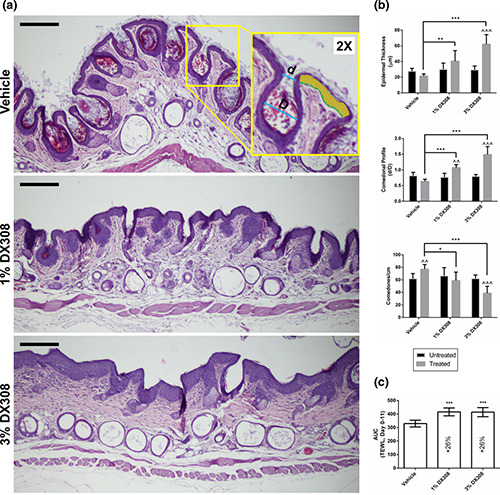

Current retinoid‐based therapies used in the treatment of disorders of keratinization and other skin disorders can result in non‐specific effects and adverse reactions. We describe DX308, a CYP26‐selective inhibitor targeting retinoid metabolism, which may address these shortcomings and result in improved patient outcomes. This preliminary study suggests that DX308 has favorable metabolic and safety profiles and reduces skin abnormalities in in vivo rhino mice.

1

**What's already known about the topic?**

- Retinoid‐based therapies are efficacious, first‐line treatments for a variety of skin disorders.
- Current retinoid‐based therapies can induce severe adverse reactions in patients via off‐target activity and over‐exposure.
- Previous retinoic acid metabolism blocking agents (RAMBA) (i.e., liarozole and talarozole) have shown improved efficacy and tolerability over traditional retinoids in clinical trials; however, they have not yet been approved, possibly due to off‐target activity.

**What does this study add?**

- DX308, a CYP26[A1/B1]‐selective RAMBA, can leverage the therapeutic potential of endogenous retinoids without high exogenous retinoid doses.
- DX308 modulates retinoid‐responsive gene expression in healthy and keratinization disorder keratinocytes, mirroring the effects of proven therapeutics, without the adverse off‐target activity.
- DX308 displays favourable target‐specificity, metabolic, and safety profiles in vitro, is well‐tolerated and reverses epidermal abnormalities in mice in vivo.

**What is the translational message?**

- The preliminary pharmacological and safety profile suggests that DX308 is a good candidate for further development as a topical therapeutic which could provide improved patient outcomes over current retinoid‐based therapies.

## 2 INTRODUCTION

Retinoid therapies are well‐established in dermatology as useful treatments for a variety of skin disorders, such as congenital ichthyosis,^1^ Darier disease (DD),^2^, ^3^, ^4^, ^5^ and others (e.g., acne and psoriasis).^6^, ^7^ Several generations of retinoids have been developed to improve target specificity and minimize the associated adverse effects.^8^ However, most available treatments risk adverse events related to metabolic autoinduction (induction of its own metabolism) and tolerance^9^, ^10^; overstimulation of retinoid biopathways from simultaneously activating retinoic acid receptors (RAR) and inhibiting retinoic acid (RA) metabolism^11^; or lack of target specificity and systemic overexposure.

A novel strategy utilizing RA metabolism blocking agents (RAMBAs)^12^ (i.e., liarozole and talarozole), which target the RA‐metabolizing CYP26 enzymes,^13^ has been investigated. RAMBAs can achieve therapeutic efficacy, without high exposure to exogenous retinoid biopathway‐stimulating compounds, by inhibiting the metabolism of endogenous RA in specific target tissues, theoretically reducing systemic overexposure and adverse effects. RAMBAs can also negate RA's problematic autoinduction of CYP26, which reduces its efficacy, requiring larger therapeutic doses leading to increased systemic exposure. By directly targeting CYP26, RAMBAs can achieve therapeutic efficacy with significantly lower, or even endogenous, RA levels. Liarozole (Figure S1) has demonstrated efficacy in several dermatological disorders^14^, ^15^, ^16^, ^17^, ^18^, ^19^, ^20^ and a trend towards better tolerability than previous retinoid therapies.^21^ Unfortunately, liarozole has also been found to display off‐target inhibition of steroid biosynthesis via CYP19 inhibition.^22^

Despite their clear potential in clinical trials, first‐generation RAMBAs ultimately never progressed, suggesting a market gap for improved RAMBA candidates. The azole moiety found on first‐generation RAMBAs is thought to contribute to their non‐specific effects by interacting with off‐target, heme‐containing enzymes such as CYP19. Based on this need, Diaz et al. developed a library of novel, azole‐free RAMBAs that specifically inhibit CYP26A1 and CYP26B1.^23^ These improved RAMBAs aim to preserve the desired therapeutic effects while minimizing off‐target activity. We previously described a CYP26B1‐selective RAMBA, DX314, which shows efficacy in vitro and in vivo.^24^

This study investigates DX308 (Figure S1), a specific inhibitor with equal affinity (IC_50_ = 51 nM) for both CYP26s found in the skin (CYP26A1 and CYP26B1), which is described in US patent US009963439B2 as example 38.^25^ In a series of in vitro assays, we characterize DX308's low potential for off‐target activity, topical irritation, and genotoxicity at the expected effective dose. We show DX308 is a good candidate for topical use, as it is stable in the skin but readily metabolized by hepatic enzymes. Additionally, both systemic and topical administration of DX308 in mice showed no evidence of apparent toxicity or hypervitaminosis A. Rhino mice, which are often used to determine the comedolytic and anti‐keratinizing activity of compounds such as retinoids,^26^, ^27^, ^28^, ^29^, ^30^ showed significant skin improvements following treatment with topical DX308. Finally, we show that DX308 modulates the expression of RA‐responsive genes and is predicted to inhibit inflammatory and immune response in healthy and keratinization disorder (i.e., congenital ichthyosis and DD) keratinocytes (KCs).

This report suggests that DX308 has potential as a safe and effective keratinization disorder therapeutic, which may present improved outcomes as an adjunct or replacement therapy for current retinoid‐based therapeutics.

## 3 MATERIALS AND METHODS

### 3.1 Nuclear receptor profiling

Nuclear receptor profiling, excluding retinoid X receptor (RXR), was previously described in detail.^24^ Briefly, profiling was done using the Steady‐Glo luciferase assay kit (Promega, E2550) in recombinant HeLa cell lines expressing the respective receptors. Compounds were treated at nine doses (*n* = 2/dose) from 0.00015–10 µM. Data were normalized to minimum and maximum controls and 4‐parameter logistic regression fitting (Figure S2) was performed in XLfit software (IDBS).

RXR activity was assessed using the human RXR reporter assay kit (IB00831‐32P, Indigo Biosciences) as per manufacturer's instructions. DX308 was tested at seven doses (*n* = 2/dose) from 0.00064–10 µM and % activity was normalized to 5 µM 9‐*cis*‐RA. Raw data was normalized and fitted in Prism (GraphPad).

### 3.2 CYP450 inhibition assays

CYP450 inhibition assays (excluding CYP19 and 1 µM CYP2C8) were performed as previously described.^31^ Substrate metabolite formation was measured by HPLC‐MS/MS. Percent of control activity was determined by comparing metabolite peak areas with and without test compound. Qualitative screening of CYP2C8 (1 µM) and CYP19 is described in detail in Supporting Information. Reference compound IC_50_ values were determined by non‐linear regression analysis using Hill equation curve fitting. Additional details are provided in Table S2.

### 3.3 In vitro metabolism assays

Estimated hepatic intrinsic clearance (CL_int_) was determined as previously described.^32^ Compound stability (% remaining) was calculated by comparing HPLC‐MS/MS peak areas at each time point to peak area at time 0. Half‐life (*T*_1/2_) was estimated by the slope of initial linear range for compound remaining versus time logarithmic curve (assuming first‐order kinetics). CL_int_ was calculated by: $\frac{0.693}{T_{1/2}*\left(\text{mg}\;\text{protein/μL}\right)}$. Additional details provided in Table S2.

Refer to Tables S1 and S2 for human skin S9 fraction metabolic stability assay methods.

### 3.4 Genotoxicity (in vitro micronucleus) assay

Genotoxicity was determined by the previously described method.^33^ Additional details are in Supporting Information and Table S2.

### 3.5 In vitro skin irritation assay

Skin irritation testing was performed using the EpiDerm kit (Epi‐200‐SIT, MatTek) per manufacturer's instructions. DX308 was prepared in polyethylene glycol 400 for topical application. Treatments (1 h) were performed in triplicate with technical duplicates and optical density was normalized to vehicle.

### 3.6 In vivo mouse safety study

Nineteen 6‐month‐old C57BL/6 mice (bred in‐house) were given intraperitoneal (i.p) injections of vehicle (hydroxypropyl‐β‐cyclodextrin), 10 or 20 mg/kg DX308 three times per week for 3 months. Mice were visually inspected for signs of hypervitaminosis A or other distress, and their weights were recorded routinely.

### 3.7 Rhino mouse study

Seventeen RHJ/LeJ rhino mice (2–3 males, 3 females per group), aged 5–7 weeks (Jackson Laboratory) received a daily application (50 µL) of vehicle (acetone) or freshly made DX308 on a 2 × 2cm area of back skin (over 11 days) as previously described.^24^ Clinical observations, body weights, transepidermal water loss (TEWL) (using Biox AquaFlux AF200 evaporimeter), and DRAIZE scoring were recorded daily. On day 12, skin was collected, processed, and stained with haematoxylin‐eosin (H&E).

Histological analysis was performed with Fiji/ImageJ^34^ software using a standardized method.^35^ Each sample was analyzed at least two different section depths. Comedonal profile (*d*/*D*) is defined as the ratio of the comedo opening (*d*) and internal diameter (*D*) (Figure 2). Epidermal thickness was measured using the ratio of epidermal area (not including the stratum corneum) and length of the underlying basal layer at a minimum of eight (mean > 19) points per subject, per group.

### 3.8 Cell culture

Normal adult KCs (NAK) and DD KCs were isolated as previously described^36^ from patient samples provided by Drs. B. Bienfait and J.S. Blairvacq (Clinique St Luc). Additional NAKs were purchased from ThermoFisher (Gibco, C‐005‐5C). Recessive X‐linked ichthyosis (RXLI) and lamellar ichthyosis (LI) KCs were provided by Northwestern University Skin Biology and Diseases Resource‐based Center (Northwestern University).

Confluent monolayer KCs were prepared as previously described^24^, ^37^ and treated for 20 h with compounds solubilized in growth media (0.1% DMSO vehicle). Reconstructed human epidermis (RHE) was produced as previously described.^24^, ^36^, ^38^ A 4‐day treatment was initiated on day 7 of tissue reconstruction. Treatments were stopped on day 11 and RHE was processed for histology or RNA isolation.

### 3.9 RNA isolation and RT‐qPCR

RNA isolation, reverse transcription, qPCR, and RNA sequencing (RNAseq) were previously described in detail.^24^ When needed, qPCR data was standardized prior to analysis using a method described by Willems et al.^39^ to correct for donor or experimental variability. Primer sequences^40^ are provided in Table S4.

### 3.10 RNAseq and bioinformatics

RNA samples were sent to the University of Colorado's Genomics and Sequence Core Facility for library preparation and sequencing on the Illumina HiSeq platform as previously described.^24^ Bioinformatics analyses were performed using Array Studio (Omicsoft) and R.^41^ Sample sequencing quality was assessed using FastQC.^42^ Raw reads were aligned to the reference genome Human.B38/Ensembl.R94 using OSA,^43^ providing 36.6–62.1 million reads per sample. Normalization and differential expression analysis were performed using a DESeq2^44^ implementation provided within Array Studio. Differentially expressed transcripts (DETs) were selected using false discovery rate (FDR)^45^ < 0.05 and |fold‐change| ≥2. Heatmaps were generated using hierarchical clustering, with Pearson correlation as distance and average linkage. Qiagen's Ingenuity Pathway Analysis (IPA) software was used for identification of biological functions/pathways enriched among the differentially expressed genes.

### 3.11 RHE histological analysis and immunostaining

RHE samples were processed as previously described^38^, ^46^ and sections were stained with H&E or prepared for immunostaining. Immunostaining was performed as previously described.^24^ Antibody details are in Table S5.

### 3.12 Statistical analysis

All statistics, excluding the otherwise described RNAseq study, were performed as indicated in the respective figures using GraphPad Prism.

## 4 RESULTS

### 4.1 DX308 has minimal off‐target nuclear receptor and CYP450 activity. DX308 is stable in the skin, readily metabolized hepatically, and is not genotoxic in vitro

Nuclear receptor profiling found that DX308 was completely inactive, or has an EC_50_ > 10 µM, in all nuclear receptors tested apart from RARα (EC_50_ = 9.04 µM) (Table 1). Inhibition assays on several major metabolic enzymes found that IC_50_s for all off‐target CYP450s investigated are >10 µM (except CYP19 which was not tested at 10 µM). CYP19 activity, which was tested in a separate manner, was not affected by 0.1 µM DX308, but saw a 61.1% reduction in the presence of 0.1 µM liarozole.

**TABLE 1:** In vitro DX308 off‐target nuclear receptor and CYP activity, hepatic and skin metabolism, and genotoxicity

Nuclear receptor activation				
Target	DX308 EC_50_ (µM)	% Activation (at 10 µM DX308)	Reference ligand	Reference ligand EC_50_ (µM)
PPARα	IA	IA	GW 7647	0.050
PPARδ	IA	IA	GW501516	0.0016
PPARγ	IA	IA	BRL49653	0.0126
LXRβ	IA	IA	GW3965	0.200
RARα	9.04	54	Tazarotenic acid	0.032
RARβ	>10	20		0.0032
RARγ	IA	IA		0.025
AR	IA	IA	BMS 564929	0.0005
GR	IA	IA	Dexamethasone	0.040
MR	IA	IA	Corticosterone	0.0004
PR	IA	IA	Progesterone	0.251
ERα	IA	IA	17β‐estradiol	0.000016
ERβ	IA	IA		0.00005
RORα^a^	>10	34	SR1078	2.512
RORγ^a^	>10	30	LYC‐55716	3.981
RXRα	>10	23	9‐*cis*‐retinoic acid	0.038
RXRβ	>10	6.7		0.059
RXRγ	>10	6.8		0.072

CYP450 inhibition			
Target	DX308 concentration (µM)	Substrate	% Inhibition
CYP1A	10	Phenacetin	−1.40
CYP2B6		Bupropion	24.6
CYP2C8		Paclitaxel	38.2
CYP2C9		Diclofenac	17.2
CYP2C19		Omeprazole	8.35
CYP2D6		Dextromethorphan	0.60
CYP3A		Midazolam	−5.95
CYP3A		Testosterone	3.55
CYP19	0.1	Androstenedione	1.50^b^
CYP2C8	1	DBOMF	22.8

Metabolism			
	*T*_1/2_ (min)	CL_int_ (µL/min/mg)	
Human liver microsomes	91–110	<115.5	
Human skin S9 fraction*	>120 (>99% remaining)		*1 & 10 µM DX308; with & without cofactors

Genotoxicity (in vitro micronucleus assay)					
Compound	Concentration (µM)	% Cytotoxicity (CBPI)	% Micronucleated cells	*p*‐value	Test results
Control‐S9	Control	0	0.5		
Mitomycin C	0.15	0.8	1.57	0.0005	+
DX308	7.8	0.4	0.63	0.2053	−
	16	−2.1	0.43	0.2856	−
	31	−2.5	0.53	0.4290	−
	63	−3.9	0.93	0.0265	−
	130	−8.1	0.91	0.0426	−
	250	−4.6	0.94	0.1819	−
	500	1.2	0.71	0.0907	−
	1000	69.2	0.34	0.1916	−

Abbreviations: CBPI, cytokinesis block proliferation index; CL_int_, intrinsic clearance; EC_50_, half maximal effective concentration; IA, Inactive; ROR, RAR‐related orphan receptor; *T*_1/2_, half‐life.

^a^ ROR receptors are constitutively active, since DX308 and respective reference ligands act as inverse agonists, % inhibition and half maximal inhibitory concentration are given in place of % activation and EC_50_.

^b^ Calculated from CYP19 activity based on decrease in metabolite formation in presence of DX308. Liarozole (0.1 nM) caused a 61.1% decrease in CYP19 activity.

* tested at 1 & 10uM DX308; with & without cofactors.

The measured *T*_1/2_ of DX308 in human liver microsomes was relatively short at 91–110 min with an estimated CL_int_ of <115.5 µL/min/mg (Table 1). In contrast, DX308 is very stable in human skin S9 fraction with, and without, cofactors (>99% remaining after 120 min). Micronucleus assay was negative for genotoxicity at ≤1000 µM, and no cytotoxicity (based on CBPI index) was reported at ≤500 µM DX308 (Table 1).

### 4.2 Topical DX308 shows no evidence of skin irritation in in vitro 3D epidermis and systemic administration in mice shows no apparent toxicity

**FIGURE 1:**
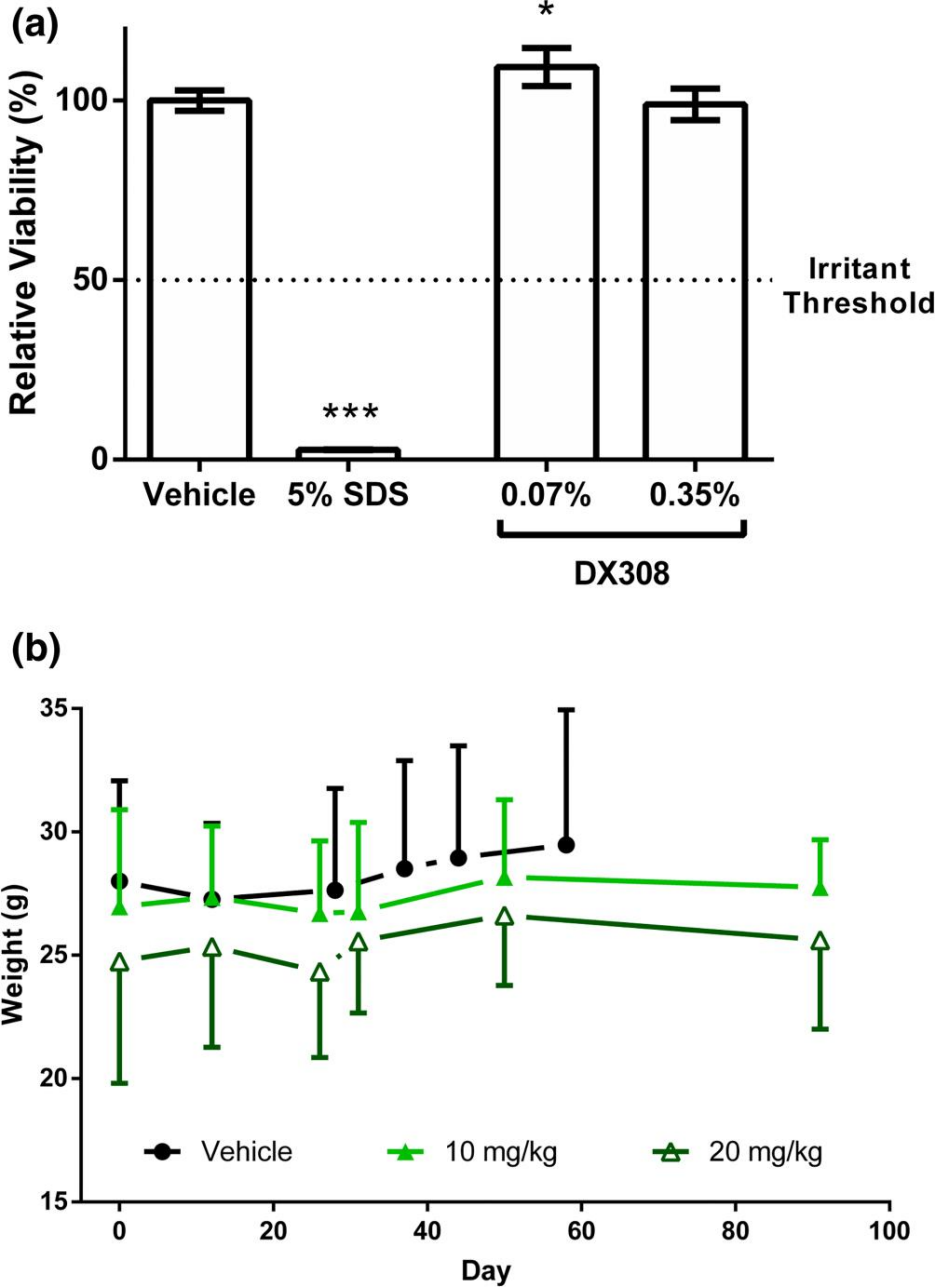
Topical in vitro skin irritation testing and long‐term systemic in vivo effect on mouse weights. (a) In vitro skin irritation test in reconstructed human epidermis treated with topical DX308. Dosage of 0.07%–0.35% corresponds to approximately 2–10 mM (*n* = 3 per group; mean ± SD; *p ≤ 0.05, ***p ≤ 0.001; one‐way ANOVA with Dunnett's correction for multiple comparisons vs. vehicle control). (b) Weight of mice injected 3× per week with intraperitoneal DX308. SD shown in single direction to maintain figure clarity (*n* = 6–7 per group, mean ± SD). SD, standard deviation; SDS, sodium dodecyl sulphate

Topically applied DX308 (0.07%–0.35%; 1.95–9.74 mM) in in vitro human 3D epidermis models, did not meet the manufacturer defined threshold for skin irritation and did not result in any decrease in cellular viability relative to control (Figure 1a).

Mice administered 10 or 20 mg/kg i.p. DX308, given three times/week during a 3‐month period, displayed no weight loss (Figure 1b) or apparent signs of toxicity or hypervitaminosis A (i.e., no spontaneous bone fractures, hair loss or bleeding). Linear regression analysis of the weights reveals no significant differences in the trends (slope) of any treatment (*p* = 0.8161) and neither treatment displays a non‐zero slope (*p* ≥ 0.3658), indicating no significant effect on body weight.

### 4.3 DX308 reverses abnormal morphology, induces comedolysis, and epidermal thickening in rhino mice

**FIGURE 2:**
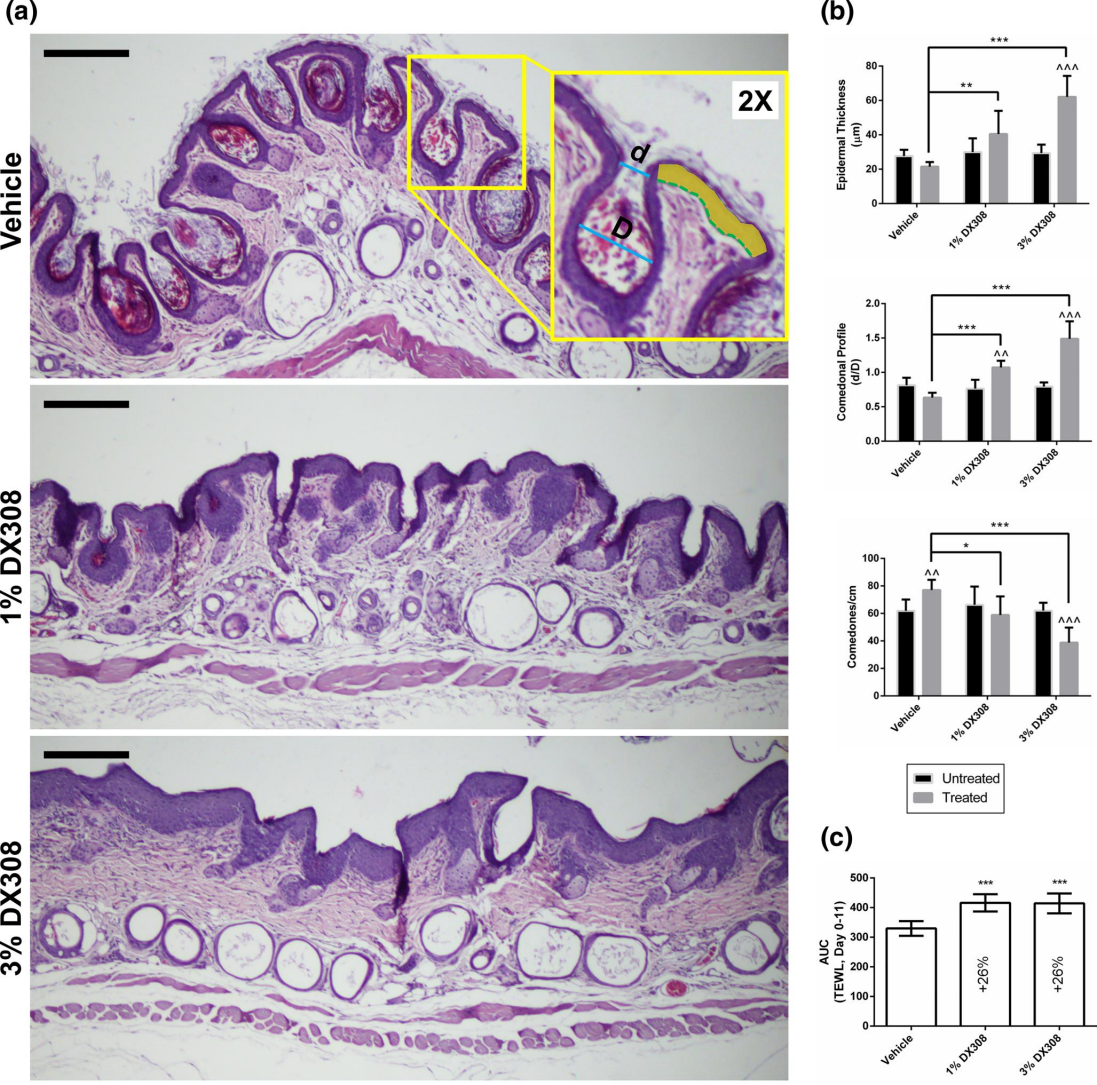
Efficacy of topical DX308 in rhino mouse model. (a) Representative image of H&E‐stained rhino mouse skin following treatment. Yellow inlay describes how comedonal profile (ratio of the comedo opening [d] to inner diameter [D]) and epidermal thickness (area of epidermis [yellow] divided by length of underlying basal layer [dotted green line]) were measured. Scale bars = 200 µm. (b) Treatment‐induced changes in epidermal thickness, comedonal profile, and comedones/cm are shown. Untreated samples are subject‐paired back tissue which received no vehicle or treatment (*n* = 5–6 mice per group; mean +  SD; *p ≤ 0.05, **p ≤ 0.01, ***p ≤ 0.001; two‐way repeated measures [subject‐paired] ANOVA with Bonferroni's correction vs. *vehicle or ^untreated skin). (c) AUC of TEWL over treatment period (*n* = 5–6 mice per group; mean ± SD; ***p ≤ 0.001; one‐way ANOVA with Dunnett's correction vs. vehicle). AUC, area under the curve; SD, standard deviation; TEWL, transepidermal water loss

Morphological skin anomalies were greatly reduced in rhino mice treated with topical DX308 (Figure 2a). Semi‐quantitative histological analysis found that DX308 induces epidermal thickening (89%–189% increase), an increase in comedonal profile (indicative of comedolysis) (69%–134% increase), and a significant decrease in comedones/cm (29%–41% decrease) (Figure 2b, Table 2). These effects scale with the dose are given. DX308‐treated mice also had a significantly larger TEWL (26% increase in AUC) relative to vehicle during the treatment period (Figure 2c). There were no instances of abnormal behaviour, adverse skin reactions, or effect on DRAIZE scoring or body weight of the mice during the study (Table S3).

**TABLE 2:** Effect of topical DX308 treatment on rhino mouse skin

	Epidermal thickness	Comedonal profile	Comedones/cm
1% DX308	+89%	+69%	−29%
3% DX308	+189%	+134%	−41%

*Note*: % change of treated groups relative to vehicle controls.

### 4.4 DX308 modulates the expression of retinoid‐responsive genes in reconstructed human epidermis and is predicted to inhibit inflammatory and immune response in skin

**FIGURE 3:**
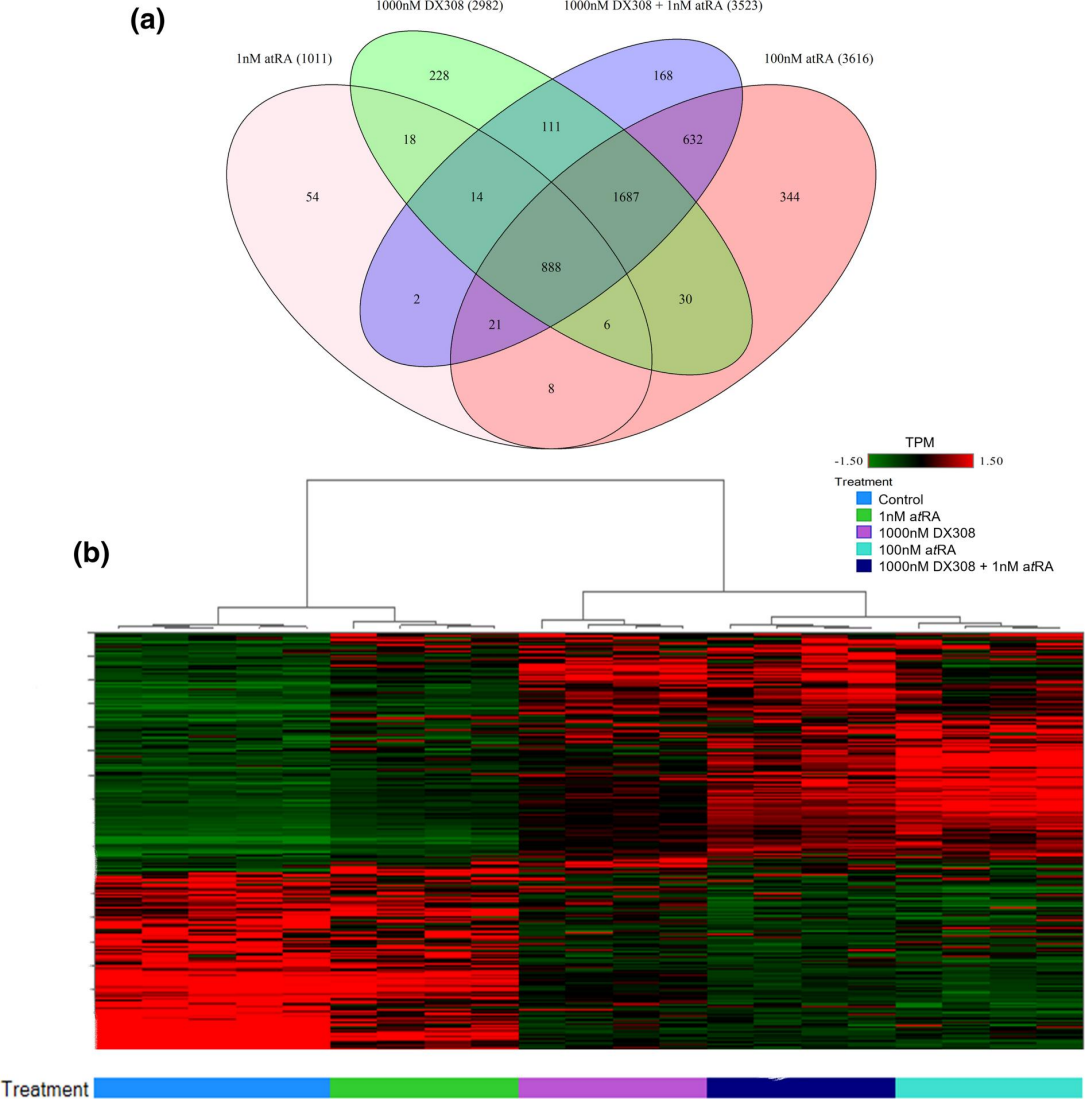
RNA sequencing of healthy reconstructed human epidermis treated with DX308 and a*t*RA. (a) Venn diagram of overlap in significantly DETs by treatment (values represent number of significantly expressed transcripts per group). (b) Hierarchical clustering of all significantly DETs. Expression shown in standardized TPM. Significantly DETs are those with a |fold‐change| ≥2 and FDR <0.05. a*t*RA, all‐*trans* retinoic acid; DETs, differentially expressed transcripts; FDR, false discovery rate; TPM, transcripts per million

Analysis of gene expression changes in healthy RHE revealed a total of 4211 DETs across all groups (FDR ≤0.05 and |fold‐change| ≥ 2). Significant overlap (3228 transcripts) is observed between the high dose (100 nM) all‐*trans* RA (a*t*RA) and the DX308 co‐treated with low dose (1 nM) a*t*RA groups, with nearly 20% of those overlapping DETs not appearing in the DX308‐alone or low dose a*t*RA‐alone groups (Figure 3a). Hierarchical clustering of all DETs by standardized transcripts per million shows tight clustering of all replicates within their respective treatment groups (Figure 3b). 100 nM a*t*RA and DX308 + 1 nM a*t*RA show the strongest correlation, while control and 1 nM a*t*RA are also tightly clustered together but as expected, form an isolated branch from the other treatment groups. Cluster analysis of epidermal differentiation complex^47^ genes (Figure S3a) shows a very similar clustering pattern. Clustering of genes involved in keratinization (Figure S3b) also follows the same pattern with one notable exception, DX308 alone shows more correlation with the control and 1 nM a*t*RA groups, than it does with the higher dose a*t*RA and DX308 + 1 nM a*t*RA groups.

**FIGURE 4:**
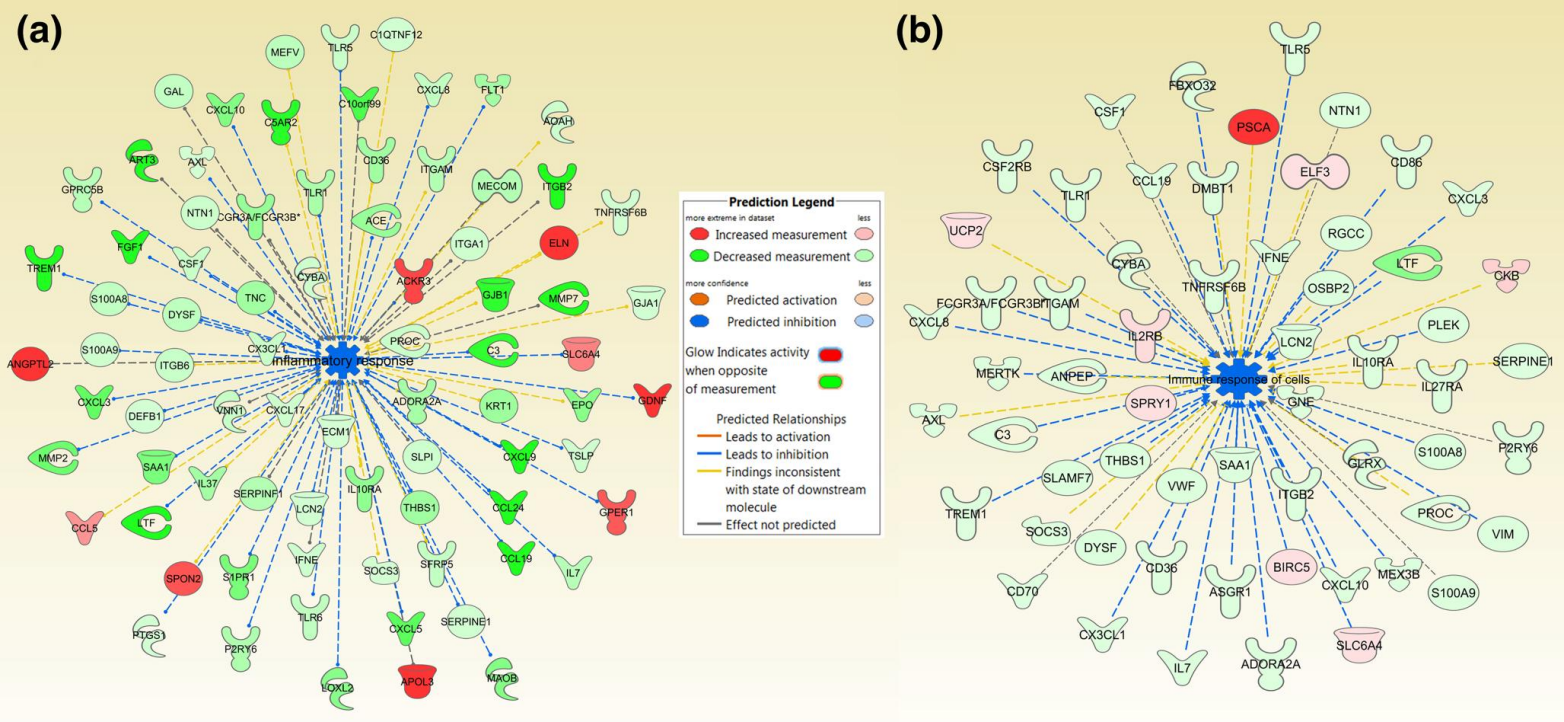
Predicted inhibition of inflammatory and immune response pathways in healthy reconstructed human epidermis treated with DX308 and a*t*RA. Ingenuity pathway analysis of all significantly differentially expressed transcripts (|fold‐change| ≥2 and FDR <0.05) revealed a predicted inhibition of (a) inflammatory and (b) immune response by 1000 nM DX308 cotreated with 1 nM a*t*RA. a*t*RA, all‐*trans* retinoic acid; FDR, false discovery rate

IPA software was used to perform predictive pathway analysis using all significantly DETs found in the RNAseq data. DX308 is predicted to inhibit inflammatory (Figure 4a) and immune response (Figure 4b) biopathways in human epidermis.

**FIGURE 5:**
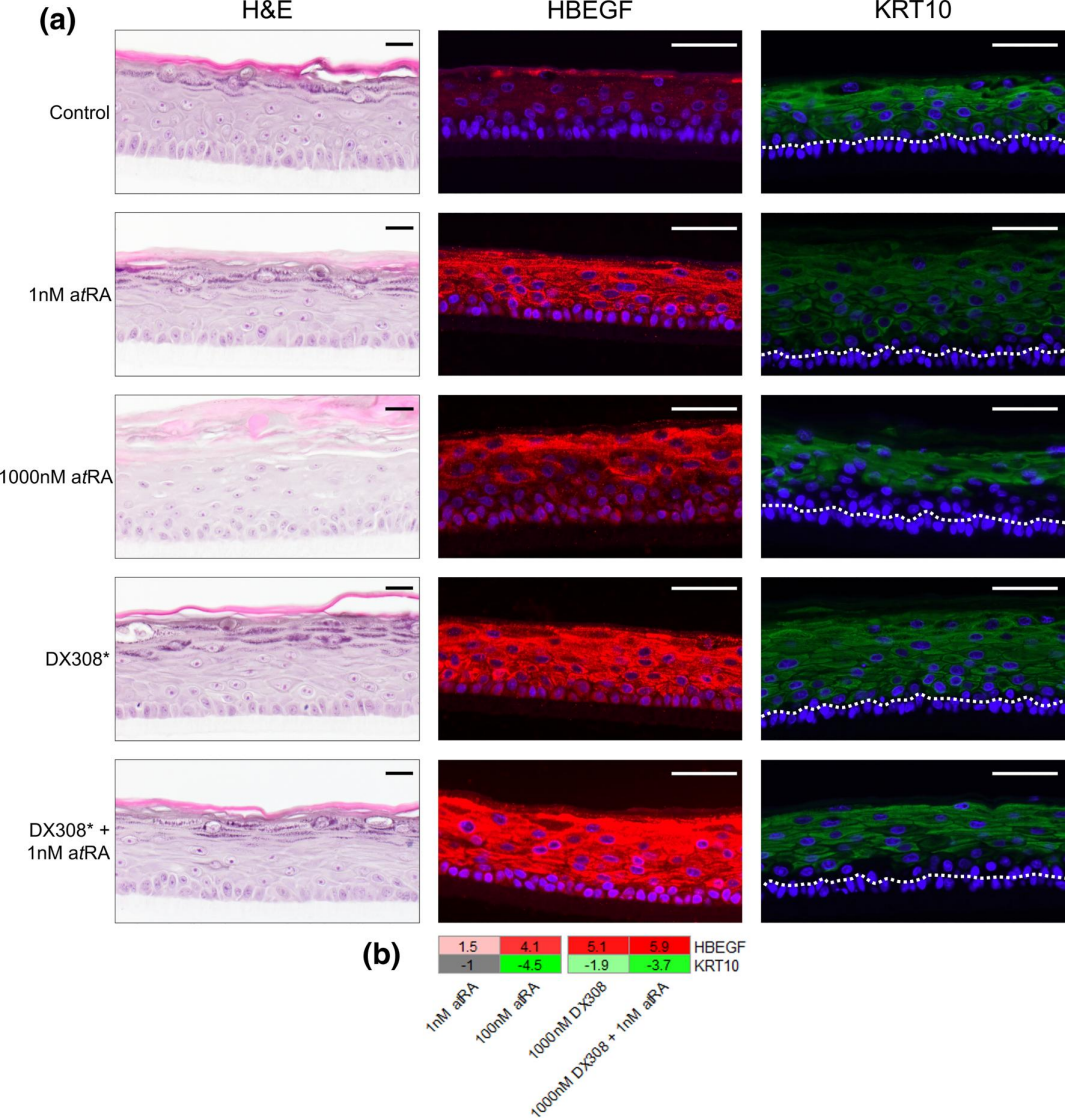
Effects of DX308 and a*t*RA on morphology and heparin‐binding EGF‐like growth factor (HBEGF) and keratin 10 (KRT10) localization in RHE. (a) H&E (left), HBEGF (middle) and KRT10 (right) staining in healthy RHE which were exposed to treatments for 4 days. Dotted white line illustrates the top of the basal keratinocyte layer. *Respective H&E and HBEGF stained RHE were treated at 100 nM DX308, KRT10 stained RHE were treated with 1000 nM DX308. Scale bars; black = 20 µm, white = 50 µm. (b) Fold‐change in HBEGF and KRT10 gene expression by RNA sequencing in healthy RHE. All non‐grey cells are significantly differentially expressed versus control (FDR ≤ 0.05). a*t*RA, all‐*trans* retinoic acid; FDR, false discovery rate; RHE, reconstructed human epidermis

Immunohistochemistry of proteins from the RA‐responsive genes, heparin‐binding EGF‐like growth factor (HBEGF) and keratin 10 (KRT10), in healthy RHE further confirm the observed gene expression patterns (Figure 5). Additionally, RA induces morphological changes in RHE, most notably a reduction in the dark keratohyalin granules indicative of the stratum granulosum and a shift in basal KCs from a characteristically columnar shape to more rounded appearance. While low dose a*t*RA alone and DX308 alone show little difference from control, DX308 in the presence of low dose a*t*RA leads to morphological changes that more closely resemble those seen from high dose a*t*RA.

### 4.5 DX308 modulates the expression of retinoid‐responsive genes across multiple healthy and keratinization disorder KCs, mimicking those of previous RAMBA liarozole

**FIGURE 6:**
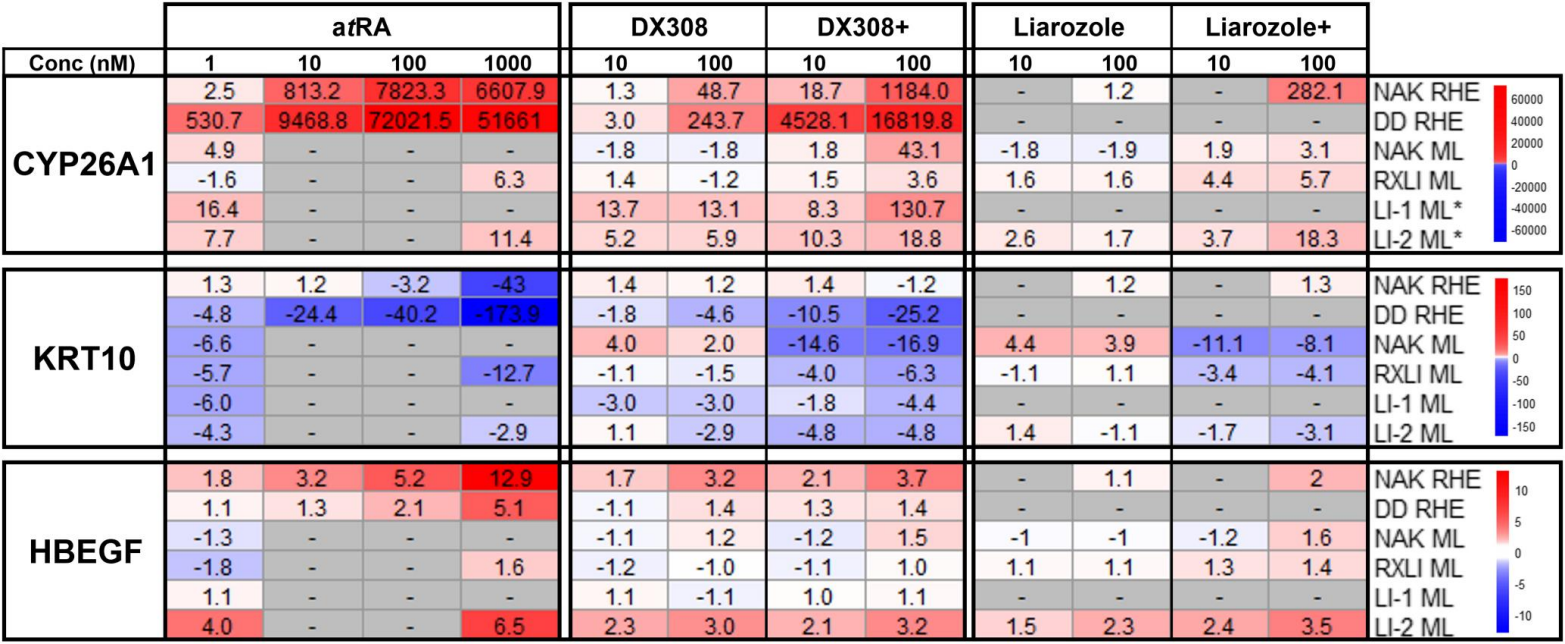
Expression of retinoid‐responsive genes CYP26A1, KRT10 and HBEGF in NAK and DD RHE; NAK, RXLI and LI monolayer keratinocytes. Values are shown as fold‐change in mRNA expression from vehicle control. *CYP26A1 mRNA was undetectable in control samples so a cycle threshold of 40 was assumed to calculate an estimate of the minimum fold‐change in expression from control for the treatment groups. NAK RHE, *n* = 3–5 (across three donors); DD RHE, *n* = 3; NAK ML, *n* = 2; RXLI ML, *n* = 3; LI‐1 ML, *n* = 3; LI‐2 ML, *n* = 3. a*t*RA, all‐*trans* retinoic acid; DD, Darier disease; DX308+, DX308 + 1 nM a*t*RA; LI, lamellar ichthyosis; Liarozole+, Liarozole + 1 nM a*t*RA; ML, monolayer; NAK, Normal Adult Keratinocytes; RHE, reconstructed human epidermis; RXLI, recessive x‐linked ichthyosis

Additional gene expression studies by RT‐qPCR in healthy (pooled results from three separate donors) and DD RHE, as well as healthy, RXLI, and LI monolayer KCs, also show modulation of RA‐responsive genes CYP26A1, KRT10 and HBEGF by a*t*RA, DX308 and the previous RAMBA liarozole (Figure 6). There is notable variability seen between pathologies, donors, and culture methods, however, expression trends are consistent. Overall, DX308 appears to induce expression changes matching or exceeding those seen from liarozole.

## 5 DISCUSSION

In this study, we establish a preclinical assessment of the pharmacological and toxicological properties of recently discovered dual CYP26A1/B1‐selective inhibitor, DX308, as a potential topical treatment for keratinization disorders and other retinoid‐responsive dermatological disorders.

In a series of preliminary in vitro experiments, we established a low risk of off‐target activity on a panel of nuclear receptors and CYP450s. Notably, we showed that DX308 does not appear to display the problematic inhibition of CYP19‐mediated steroid biosynthesis seen in the previous RAMBA liarozole. Additional in vitro toxicological profiling of potential genotoxicity and skin irritation also showed minimal risks within the effective dose range and suggested that DX308 has a favourable safety profile.

Since DX308 is being developed as a topical treatment, which minimizes systemic retinoid biopathway stimulation, it is important to ensure stability in the target tissue (human skin) and rapid metabolism upon systemic exposure to minimize off‐target tissue effects. Indeed, our data show that DX308 displays a relatively short half‐life in hepatic microsomes and good metabolic stability in skin S9 fraction.

In vivo mouse studies further reinforce DX308 as having a low risk of toxicity. Regular, high‐dose, long‐term systemic administration of DX308 did not result in weight loss or apparent signs of toxicity or hypervitaminosis A. Another study of daily topical administration over 11 days did not show any effect on mouse weight, DRAIZE scoring, or abnormal clinical observations compared to control mice.

In addition to its favourable metabolic and toxicologic profile, topical DX308 significantly improved the markedly abnormal skin of rhino mice. We showed an increase in comedonal profile, which is an indicator of comedolysis, in addition to reductions in the total number of comedones. Perhaps due to the harsh acetone vehicle used in this initial topical study, we did observe an increase in comedones/cm in vehicle‐treated skin compared to untreated skin, however, the effect of DX308 in both reversing and overcoming the increased comedones was still apparent. Currently, improved topical formulations are being developed for DX308, which are expected to be well‐tolerated, and should allow for future studies without a significant vehicle effect. DX308 also induced an expected increase in epidermal thickness and overall TEWL, effects that have been well‐documented in rhino mice treated with retinoid biopathway‐stimulating compounds.^48^, ^49^

To confirm that the activity of DX308 is mediated by its activation of retinoid biopathways, we investigated the transcriptomic profile of RHE treated with DX308 and a*t*RA. Hierarchical cluster analysis of all significant DETs found the strongest correlation between high dose a*t*RA and DX308 cotreated with low dose a*t*RA, while low dose a*t*RA alone clustered together with the vehicle control. This suggests that DX308 is potentiating the effect of low dose a*t*RA by inhibiting its metabolism and, in turn, producing an expression profile mimicking treatment with a much higher dose of a*t*RA.

Predictive pathway analysis using said RNAseq profile also suggests that DX308 may inhibit inflammatory and immune response pathways, potentially positioning DX308 as treatment of retinoid‐responsive skin diseases with inflammation, such as acne and psoriasis.

In RHE, immunostaining for HBEGF, a retinoid‐responsive indicator of KC proliferation^50^, ^51^, ^52^, ^53^ in RHE, was increased in parallel with its increased mRNA expression. Similarly, staining and expression of KRT10, an early marker of epidermal differentiation, were reduced by 1000 nM DX308 with low dose a*t*RA commensurate with the reduction seen from high dose a*t*RA. Morphological changes induced by high dose a*t*RA (decrease in keratohyalin granules/stratum granulosum and rounding of the typically columnar basal KCs) were also observed in DX308 with low dose a*t*RA, which further supports a potentiation of a*t*RA effects by CYP26 inhibition.

To further reinforce DX308's ability to modulate retinoid biopathways in multiple donors and pathologies, the expression of three retinoid‐responsive genes (CYP26A1, KRT10 and HBEGF) were investigated in healthy and keratinization disorder (including DD, RXLI and LI) RHE and monolayer KCs. Despite varying effect sizes, we saw relatively consistent expression trends across donors and pathologies. In addition to modulating these genes in a manner consistent with a*t*RA treatment, DX308 also induced expression patterns mimicking those seen with the previously investigated, well‐characterized RAMBA liarozole. Considering clinical trials showing liarozole is a well‐tolerated and effective therapeutic, in addition to DX308's lack of off‐target CYP450 activity (in contrast to liarozole), these data suggest that DX308 should perform as an effective therapeutic without the shortcomings that likely prevented FDA approval of liarozole.

Together, this study introduces and characterizes a novel CYP26[A1/B1]‐selective inhibitor, DX308, as a potential therapeutic for keratinization disorders. DX308 has a low risk of toxicity, off‐target activity, and irritation, a favourable metabolic profile for topical application, reverses abnormal skin morphology in rhino mouse skin, and modulates the expression retinoid‐responsive genes in healthy and keratinization disorder KCs.

This report provides a preliminary profile on DX308, but still requires further investigation into its detailed pharmacodynamics, pharmacokinetics, and in vivo human efficacy. Based on the data obtained in this study, DX308 is a promising candidate for further investigation as an improved keratinization disorder therapeutic and may address the shortcomings of current therapeutic strategies to improve patient outcomes.

## CONFLICT OF INTERESTS

P. Diaz is cofounder of DermaXon™ and inventor of the technology; he and The University of Montana are entitled to future royalty payments. D. Mendes, J. Kreitinger and L. Walker were employed by DermaXon™ during their contributions to the study. J.G.S. Veit was employed at DermaXon™ during a portion of this study.

## ETHICAL APPROVAL

All animal studies were approved by the institutional animal care and use committee under the National Institute of Health guidelines.

[Correction added on 24 February 2022, after first online publication: The ethical approval statement was missing and has been reinstated in this version.]

## Supporting information

Supporting Information 1

## Data Availability

Data available upon request.
