# Supplementary material for: Preclinical assessment of dual CYP26[A1/B1] inhibitor, DX308, as an improved treatment for keratinization disorders
Source: Skin Health Dis. 2021 Mar 26;1(2):e22. doi: 10.1002/ski2.22 (PMC9060145; doi:10.1002/ski2.22)
Supplement: Supplementary file 1 — Supporting Information 1 [file SKI2-1-e22-s001.docx]

# Supplementary Methods

## Qualitative screening of CYP2C8 and CYP19

The Vivid® CYP2C8 Green Screening Kit was purchased from Life Technologies (Carslbad, CA). Montelukast, estrone, and androstenedione were purchased from Cayman Chemical (Ann Arbor, MI). Human CYP19 + P450 Reductase Supersomes™ was purchased from Corning (Woburn, MA). Cortisone was purchased from MP Biomedicals, LLC (Solon, OH). β-Nicotinamide adenine dinucleotide 2′-phosphate reduced tetrasodium salt hydrate (NAPDH) was purchased from Sigma Aldrich (St. Louis, MO). Liarozole dihydrochloride was purchased from Tocris Bioscience (Bristol, UK). Black 96-well Costar plates were purchased from Thermo Fisher Scientific (Waltham, MA). HPLC-grade solvents, acetonitrile and dimethyl sulfoxide (DMSO) were purchased from Fisher Chemical (Fair Lawn, NJ) and formic acid from Mallinckrodt Chemicals (Phillipsburg, NJ).

A kinetic reaction was carried out for CYP2C8 screening in triplicate according to the Vivid® CYP450 Screening Kit manual protocol (MAN0003095). Briefly, a master pre-mix composed of CYP2C8 Baculosomes®, regeneration system of human P450 reductase/human cytochrome b5, and reaction buffer was prepared before adding 50 μL to each well in a black 96-well plate. Test compounds, positive inhibition control, and solvent controls were prepared as a 2.5X concentration with DMSO in reaction buffer of which 40 μL was dispensed into the 96-well plate. Concentration (X) of test compounds and controls was 1 μm, 0.1% DMSO in a final 100 μL volume reaction mixture (including Vivid® DBOMF substrate and NADP^+^) per well. The test samples were pre-incubated at 37 °C for 15 min in the fluorescent microplate reader before adding a 10 μL mixture of substrate and NADP^+^. Immediately, the plate was read at 2 min intervals over a 30 min time period at 37 °C. The raw data in relative fluorescent units (RFU) was analyzed according to the kinetic assay mode as outlined in the published protocol.

*In vitro* CYP19 screening was carried out in triplicate using a modified HPLC-MS/MS protocol based on methodology provided by Admescope Ltd. DMSO stock solutions of test compounds, positive control liarozole, androstenedione substrate and solvent control were diluted with 100 mM phosphate buffer (pH 7.4) such that DMSO content in final assay conditions was 1% in a volume of 200 μL. Briefly, to a microfuge tube was added 1 μM substrate, 1 mM NAPDH, and 0.1-1 μM test compounds (final concentrations after enzyme addition) in phosphate buffer before pre-incubating for 2 min at 37 °C. CYP19/CYP450 reductase Supersomes™ enzyme (50 μL, 1.5 pmol/mL) was added to each tube, gently mixed, then incubated at 37 °C for 30 min. The reaction was quenched with an equal volume of acetonitrile containing cortisone internal standard (100 nM). The tubes were vortexed and centrifuged at 13,200 x *g* for 10 min followed by aliquoting supernatant to LCMS vials for immediate analysis.

Estrone metabolite formation was measured using a HPLC-MS/MS analytical platform. Liquid chromatography was performed using a Gemini NX-C18 column (3 µm, 110Å, 50 x 4.6 mm; Phenomenex) equilibrated at 40 °C in an Agilent 1200 series HPLC system. The sample injection volume was 6 µL. Chromatographic separation was conducted using a mobile phase of (A) 0.1% formic acid and (B) acetonitrile. Initial mobile phase conditions with a flow rate of 0.4 mL/min were 50% A and B then a gradient method was applied as follows: 50 - 77% B (0.00 - 2.00 min, 77 - 78% B (2.00 - 3.50 min), 78 - 90% B (3.50 - 5.00 min), then 90 - 50% B (5.00 - 7.00 min) and re-equilibration at 50% B for 1 min, for a total run time of 8 min. For mass analysis, the HPLC was coupled to an Agilent 6460 triple-quadrupole mass spectrometer with an AJS electrospray ionization (ESI) source set in positive mode. The first 1.0 min of the run was directed to waste to decrease ion source contamination. Generic source parameters for all analyses were 45 psi nebulizer gas, 325 °C carrier gas at 10 L/min, 350 °C sheath gas at 12 L/min, capillary voltage 1500 V and nozzle voltage of 500 V. The optimal collision energy was determined as 22 V with fragmentor voltages of 118 V for cortisone and androstenedione and 80 V for estrone. Multiple reaction monitoring (MRM) transitions were then monitored in positive ion mode as follows: cortisone (m/z 361.1 → 163.1), androstenedione (m/z 287.2 → 97.1), and estrone (m/z 271.1 → 133.1).  Data was analyzed using Agilent Mass Hunter QQQ Quantitative Analysis B.07.01 software. Estrone concentration values derived from a calibration curve (linear regression) were then substituted in Equation (1) to determine remaining %Activity where X is the concentration of estrone (ng/mL) in presence of test compound and A is the concentration of estrone (ng/mL) in the solvent control (1% DMSO).

%𝐴𝑐𝑡𝑖𝑣𝑖𝑡𝑦 = $\left( \frac{X}{A} \right)*100$ (1)

Quality control (QC) samples prepared at 25 nM, 100, nM and 200 nM validated error in the calibration curve as less than 10% in between runs of sample analyses.

## Genotoxicity (in vitro micronucleus) assay

Marginally positive results (-/+) are defined by the % micronucleated cell value being significantly higher than control (p<0.05) and at least 2-fold higher. Positive results (+) are significantly higher (p<0.05) and at least 3-fold higher than control. A "scorable" cell is a bi-nucleated cell contained within the field boundary with nuclei of similar size (maximum area ratio of large to small nucleus is <1.5). Cytokinesis Block Proliferation Index (CBPI) % cytotoxicity uses a modified version of the CBPI (CBPI of 1 = 100% cytotoxicity) and calculated as follows: $Cytotoxicity \left( \% \right)=100-(\frac{{CBPI}_{t}-1}{{CBPI}_{c}-1}*100)$; (t = treated, c = control); $CBPI=\frac{MoNC+2*MuNC}{total \# of cells}$; (MoNC = number of mononuleated cells, MuNC = number of multinucleated cells, since most MuNC cells are binucleated). % micronucleated cells is the % of scorable cells with at least one micronucleus and a micronucleus vs average nuclei radius ratio of ≤0.33. Additional details provided in **Table S2**.

| **Table S1:** Experimental methodology for human skin S9 fraction metabolic assay | | | | | | | | | |
| --- | --- | --- | --- | --- | --- | --- | --- | --- | --- |
| **Chemicals** | | | | | | | | | |
| HPLC grade methanol and acetonitrile: Merck (Darmstadt, Germany). HPLC grade formic acid, acetic acid, ammonium acetate and ammonium formate: BDH Laboratory Supplies (Poole, UK). Other chemicals: Sigma Aldrich (Helsinki, Finland), the highest purity available. Water was in-house freshly prepared with a Direct-Q3 (Millipore Oy, Espoo, Finland) purification system and UP grade (ultra pure, 18.2 MΩ). | | | | | | | | | |
|  | | | | | | | | | |
| **Incubation** | | | | | | | | | |
| Enzyme source / supplier | | | | Pooled skin S9 / Bioreclamation IVT, See Appendix I for a detailed description | | | | | |
| Species / strain | | | | Human / mixed gender | | | | | |
| Conditions in the final incubation: | | | | | | | | | |
| Incubation volume | | | | 300 µl in eppendorf-tubes | | | | | |
| Buffer | | | | 0.1 M phosphate buffer, pH 7.4 | | | | | |
| Protein content | | | | 2.0 mg/ml | | | | | |
| Cofactors & concentrations | | | | NADPH + UDPGA + PAPS + GSH + AcetylCoA (1 mM) | | | | | |
| Test compound | | | | 1 µM, 10 µM (stock solution in DMSO) | | | | | |
| DMSO content in incubation | | | | 0.5% | | | | | |
| Time points | | | | 0, 30, 60, 90 and 120 min with cofactors, 0 and 120 min without cofactors | | | | | |
| Preincubation time | | | | 2 min@ 37^o^C | | | | | |
| Temperature | | | | 37^o^C | | | | | |
| Shaking | | | | 600 rpm | | | | | |
| Reaction started by | | | | Addition of study compound | | | | | |
| Sampling volume | | | | 50 µl | | | | | |
| Termination of incubations | | | | 2-fold volume of acetonitrile | | | | | |
| Storage | | | | –20^o^C until analysis | | | | | |
| Control compounds | | | | Trandolapril and 4-methylumbelliferone | | | | | |
|  | | | | | | | | | |
| **Sample preparation** | | | | | | | | | |
| The samples were thawed at room temperature (RT), shaken and centrifuged for 10 min at 13,000 × g (Heraeus Pico 17 centrifuge, room temperature), and pipetted to Waters 96-well UPLC-plate for analysis. | | | | | | | | | |
|  | | | | | | | | | |
| **Liquid chromatography-mass spectrometry** | | | | | | | | | |
| Instrumentation | | Waters Acquity UPLC + Q-Exactive Focus Orbitrap mass spectrometer + PDA detector | | | | | | | |
| Column | | Waters Acquity HSS T3 (2.1 × 50 mm, 1.7 µm) column with guard filter | | | | | | | |
| Polarity | | ESI+ | | | | | | | |
| Sheath Gas | | nitrogen 50 units | | | | | | | |
| Auxiliary Gas | | nitrogen 10 units | | | | | | | |
| Sweep Gas | | nitrogen 4 units | | | | | | | |
| Capillary voltage | | 4000 V | | | | | | | |
| Capillary Temp | | 320 (°C) | | | | | | | |
| Auxiliary Gas Temp | | 500 (°C) | | | | | | | |
| Mass range | | m/z 70 – 1000 | | | | | | | |
| Acquisition time | | 7 Hz for full scan, IT 100 ms for DDI MS/MS | | | | | | | |
| AGC target | | 1E6 | | | | | | | |
| Max IT | | 100 ms | | | | | | | |
| Collision energy | | 30 + 50 eV (data dependent, based on inclusion list) | | | | | | | |
| Resolution | | 35 000 (FWHM @ m/z 200) for full scan, 17 500 for MS/MS | | | | | | | |
| Calibration | | External | | | | | | | |
| PDA-range | | 210 – 500 nm | | | | | | | |
| Software | | Thermo Xcalibur 3.0.63 | | | | | | | |
| Other information | | First 0.5 min of the run was directed into waste by using a divert valve to decrease the ion source contamination by early eluting matrix constituents. | | | | | | | |
| Time | | Flow | | | A% | | B% | | |
| 0.00 min | | 0.500 ml/min | | | 95 | | 5 | | |
| 0.50 min | | 0.500 ml/min | | | 95 | | 5 | | |
| 3.00 min | | 0.500 ml/min | | | 10 | | 90 | | |
| 3.50 min | | 0.500 ml/min | | | 2 | | 98 | | |
| 4.00 min | | 0.500 ml/min | | | 2 | | 98 | | |
| 5.00 min | | 0.500 ml/min | | | 95 | | 5 | | |
| Column oven temperature 35 (°C) | | | | | | | | | |
| Injection volume 4 (µl) | | | | | | | | | |
| Ion chromatograms were extracted from the total ion chromatograms using calculated monoisotopic accurate masses with 5 mDa window. | | | | | | | | | |
|  | | | | | | | | | |
| **Characteristics of human skin S9** | | | | | | | | | |
| Mixed gender human skin S9, pooled | | | | | | | | | |
| BioreclamationIVT product number S03405 | | | | | | | | | |
| Lot number BFP | | | | | | | | | |
| **Enzyme** | **Assay** | | | | | | | **Activity (ρmol/mg/min)** | |
| UGT | 7-OH-coumarin glucuronidation | | | | | | | 0 | |
| CYP3A4 | Testosterone-6β-hydroxylase | | | | | | | 2.45 | |
|  | | | | | | | | | |
| **Metabolite formation from control compounds at 60min** | | | | | | | | | |
| **Control compound** | | | **Concentration (µM)** | | | **Metabolite** | | | **Formation, % from 0min parent** |
| Trandolapril | | | 10 | | | Ester hydrolysis | | | 7.8 |
| 4-Methylumbelliferone | | | 10 | | | Sulfo-conjugation | | | 8.1 |

| **Table S2:** Additional methodological details regarding *in vitro* CYP activity, skin and hepatic metabolism, and genotoxicity profiling | | | | | | | | | | | | | | | | | |
| --- | --- | --- | --- | --- | --- | --- | --- | --- | --- | --- | --- | --- | --- | --- | --- | --- | --- |
| **Metabolism Assays** | | | | | | | | | | | | | | | | | |
| **Human Liver Microsomes** | | **Source** | **Reference Compound** | | **Concentration (nM)** | | **T_1/2_ (min)** | | | **CL_int_ (µL/min/mg)** | | | **Incubation** | | **Measured Component** | | **Method** |
|  |  |  |  |  |  |  | **1** | | **2** |  |  |  |  |  |  |  |  |
|  |  | **human liver microsomes (0.1mg/mL)** | **imipramine** | | **100** | | **96.1** | | **101** | **<115.5** | | | **0, 15, 30, 45, 60min; 37°C** | | **test compound** | | **HPLC-MS/MS** |
|  |  |  | **imipramine** | | **100** | | **148.8** | | **191.8** | **<115.5** | | |  |  |  |  |  |
|  |  |  | **propranolol** | | **100** | | **95.1** | | **106.3** | **<115.5** | | |  |  |  |  |  |
|  |  |  | **propranolol** | | **100** | | **310.7** | | **307.5** | **<115.5** | | |  |  |  |  |  |
|  |  |  | **terfenadine** | | **100** | | **25.2** | | **25.7** | **272.3** | | |  |  |  |  |  |
|  |  |  | **terfenadine** | | **100** | | **18.5** | | **17.2** | **388.7** | | |  |  |  |  |  |
|  |  |  | **verapamil** | | **100** | | **28.5** | | **27.1** | **249.6** | | |  |  |  |  |  |
|  |  |  | **verapamil** | | **100** | | **27.9** | | **28** | **247.6** | | |  |  |  |  |  |
|  | |  |  | |  | |  | |  |  | | |  | |  | |  |
| **Human Skin S9 Fraction** | | **Source** | **Reference Compound** | | **Concentration (nM)** | | **Metabolite formation (% from parent)** | | | | | | **Incubation** | | **Measured Component** | | **Method** |
|  |  | **mixed gender human skin S9, pooled** | **Trandolapril** | | **1x10^4^** | | **7.8** | | | | | | **0, 30, 60, 90, 120min; 37°C** | | **ester hydrolysis** | | **UPLC/QE-orbitrap-MS** |
|  |  |  | **4-Methylumbelliferone** | | **1x10^4^** | | **8.1** | | | | | |  |  | **sulfo-conjugation** | |  |
|  | |  |  | |  | |  | |  | |  | |  | |  | |  |
| **CYP Inhibition Assays** | | | | | | | | | | | | | | | | | |
| **Target** | **Source** | | **Reference Compound** | **IC_50_ (nM)** | | **nH** | | **Substrate** | | **Substrate Concentration (nM)** | | | **Incubation** | | **Measured Component** | | **Method** |
| **CYP1A** | **human liver microsomes (0.1mg/mL)** | | **furafylline** | **2.2x10^4^** | | **0.7** | | **phenacetin** | | **1x10^4^** | | | **10min; 37°C** | | **acetaminophen** | | **HPLC-MS/MS** |
| **CYP2B6** |  |  | **clopidogrel** | **370** | | **1.3** | | **bupropion** | | **1x10^5^** | | |  |  | **hydroxybupropion** | |  |
| **CYP2C8** |  |  | **montelukast** | **200** | | **0.6** | | **paclitaxel** | | **1x10^4^** | | |  |  | **6α-hydroxypaclitaxel** | |  |
| **CYP2C9** |  |  | **sulfaphenazole** | **220** | | **0.8** | | **diclofenac** | | **1x10^4^** | | |  |  | **4'-hydroxydiclofenac** | |  |
| **CYP2C19** |  |  | **oxybutynin** | **4000** | | **0.8** | | **omeprazole** | | **500** | | |  |  | **5-hydroxyomeprazole** | |  |
| **CYP2D6** |  |  | **quinidine** | **100** | | **0.8** | | **dextromethorphan** | | **5000** | | |  |  | **dextrorphan** | |  |
| **CYP3A** |  |  | **ketoconazole** | **200** | | **1.7** | | **midazolam** | | **5000** | | |  |  | **1-hydroxymidazolam** | |  |
| **CYP3A** |  |  | **ketoconazole** | **300** | | **1.6** | | **testosterone** | | **5x10^4^** | | |  |  | **6β-hydroxytestosterone** | |  |
| **Target** | **Source** | | **Reference Compound** | **Reference Concentration (nM)** | | **% Inhibition** | | **Substrate** | | **Substrate Concentration (nM)** | | | **Incubation** | | **Measured Component** | | **Method** |
| **CYP19** | **CYP19 Supersomes™ (Corning 456260)** | | **liarozole** | **1000** | | **83.8** | | **androstenedione** | | **1000** | | | **30min; 37°C** | | **estrone** | | **HPLC-MS/MS** |
| **CYP2C8** | **Vivid™ CYP2C8 kit (Invitrogen PV6141)** | | **montelukast** | **1000** | | **100.0** | | **DBOMF** | | **1000** | | | **measure every 2min for 30min; 37°C** | | **DBOMF Green** | | **fluorimetry** |
|  |  | |  |  | |  | |  | | |  |  | |  | |  | |
| **Genotoxicity (*in vitro* micronucleus test)** | | | | | | | | | | | | | | | | | |
|  | **Source** | | **Technique** | | | | | | | | | **Incubation** | | **Measured Component** | | **Method** | |
|  | **CHO-K1 cell line** | | **high content analysis (HCA)** | | | | | | | | | **24hr, 37°C** | | **micronucleated cells** | | **fluorescent image analysis** | |
| CL_int_, intrinsic clearance; EC_50_, half maximal effective concentration; IC_50_, half maximal inhibitory concentration; nH, hill coefficient; T_1/2_, half-life. | | | | | | | | | | | | | | | | | |

| **Table S3:** Sex, body weights, DRAIZE scoring, and clinical observations of rhino mice | | | | | | | | | | | |
| --- | --- | --- | --- | --- | --- | --- | --- | --- | --- | --- | --- |
| **Treatment** | **Sex** | **Weight (g)** | | | **DRAIZE Scoring** | | | | | | **Clinical Observations** |
|  |  | **Day 0** | **Day 7** | **Day 12** | **Day 0** | | **Day 7** | | **Day 12** | |  |
|  |  |  |  |  | **Erythema** | **Edema** | **Erythema** | **Edema** | **Erythema** | **Edema** |  |
| Vehicle | M | 24.5 | 20.8 | 21.9 | 0 | 0 | 0 | 0 | 0 | 0 | NAO (day 0-5); slight dry skin on upper back (day 6); dry skin on upper back (day 7-8); slight dry skin on upper back (day 9-11); NAO (day 12) |
|  | M | 24.4 | 25.9 | 25.2 | 0 | 0 | 0 | 0 | 0 | 0 | NAO (day 0-8); very slight dry skin on upper back (day 9); NAO (day 10-12) |
|  | M | 18.5 | 19.1 | 19.8 | 0 | 0 | 0 | 0 | 0 | 0 | NAO (day 0-5); slight dry skin on upper back (day 6-7); NAO (day 8); two small bite marks at base of tail (day 9-11); NAO (day 12) |
|  | F | 19.3 | 19.6 | 19.7 | 0 | 0 | 0 | 0 | 0 | 0 | NAO (day 0-6); slight dry skin on upper back (day 7-8); NAO (day 9); slight dry skin on upper back (day 10-11); NAO (day 12) |
|  | F | 21.9 | 21.8 | 23.2 | 0 | 0 | 0 | 0 | 0 | 0 | NAO (day 0-7); slight dry skin on upper back (day 8); NAO (day 9-12) |
| 1% DX308 | M | 25.7 | 26.5 | 26.3 | 0 | 0 | 0 | 0 | 0 | 0 | NAO (day 0-5); mild dry skin over whole back (day 6-7); dry skin over whole back (day 8); mild dry skin over whole back (day 9-11); NAO (day 12) |
|  | M | 23.3 | 23.1 | 24.1 | 0 | 0 | 0 | 0 | 0 | 0 | NAO (day 0-6); slight dry skin on whole back (day 7); slight dry skin on upper back (day 8); NAO (day 9-12) |
|  | M | 21.4 | 21.6 | 21.5 | 0 | 0 | 0 | 0 | 0 | 0 | NAO (day 0-6); dry skin on upper back (day 7); slight dry skin on upper back (day 8); NAO (day 9-12) |
|  | F | 21.8 | 23.3 | 23 | 0 | 0 | 0 | 0 | 0 | 0 | NAO (day 0-6); dry skin on upper back (day 7); slight dry skin on upper back (day 8); NAO (day 9-12) |
|  | F | 22.1 | 22.2 | 22.4 | 0 | 0 | 0 | 0 | 0 | 0 | NAO (day 0-6); dry skin on upper back (day 7); slight dry skin on upper back (day 8); NAO (day 9-12) |
|  | F | 20.3 | 21.5 | 22 | 0 | 0 | 0 | 0 | 0 | 0 | NAO (day 0-6); slight dry skin on upper back (day 7-8); NAO (day 9); slight dry skin on upper back (day 10-12) |
| 3% DX308 | M | 25.1 | 25.3 | 25 | 0 | 0 | 0 | 0 | 0 | 0 | NAO (day 0-6); dry skin on whole back (day 7); dry skin on upper back (day 8-12) |
|  | M | 24.4 | 24.7 | 23.6 | 0 | 0 | 0 | 0 | 0 | 0 | NAO (day 0-5); slight dry skin on whole back (day 6); dry skin on whole back (day 7-8); slight dry skin on upper back (day 9-12) |
|  | M | 22.5 | 22.9 | 23.9 | 0 | 0 | 0 | 0 | 0 | 0 | NAO (day 0-6); dry skin on upper back (day 7); slight dry skin on whole back (day 8); slight dry skin on upper back (day 9-12) |
|  | F | 21.4 | 22.4 | 22.1 | 0 | 0 | 0 | 0 | 0 | 0 | NAO (day 0-6); dry skin on whole back (day 7); slight dry skin over whole back (day 8-12) |
|  | F | 21.5 | 22.5 | 22.6 | 0 | 0 | 0 | 0 | 0 | 0 | NAO (day 0-5); slight dry skin on upper back (day 6-8); NAO (day 9-11); slight dry skin on whole back (day 12) |
|  | F | 21.3 | 22.1 | 21.9 | 0 | 0 | 0 | 0 | 0 | 0 | NAO (day 0-5); slight dry skin over whole back (day 6-8); NAO (day 9-11); slight dry skin on upper back (day 12) |
| F, female; M, male; NAO, No Abnormal Observations | | | | | | | | | | | |

| **Table S4:** Primer sequences used for RT-qPCR | | |
| --- | --- | --- |
| **Target** | **Forward** | **Reverse** |
| 36B4 | ATCAACGGGTACAAACGAGTC | CAGATGGATCAGCCAAGAAGG |
| RPL13a | CTCAAGGTCGTGCGTCTGAA | TGGCTGTCACTGCCTGGTACT |
| HBEGF | TGGCCCTCCACTCCTCATC | GGGTCACAGAACCATCCTAGCT |
| KRT10 | ATCGATGACCTTAAAAATCAGATTCTC | GCAGAGCTACCTCATTCTCATAC |
| CYP26A1 | GGGAGAGCGGCTGGACAT | TCCAAAGAGGAGTTCGGTTGA |

| **Table S5:** Antibodies used in study | | | |
| --- | --- | --- | --- |
| **Target** | **Species** | **Dilution** | **Source** |
| HBEGF | Goat | 1:200 | Calbiochem, PC319L |
| KRT10 | Mouse | 1:100 | DAKO, M7002 |
| 2°, Alexa Fluor 488 (anti-mouse) | Goat | 1:1000 | Life Technologies, A11001 |
| 2°, Alexa Fluor 568 (anti-goat) | Donkey | 1:200 | Life Technologies, A11057 |
| Hoechst 33258, nuclear stain | - | 1:100 | Life Technologies, H3569 |


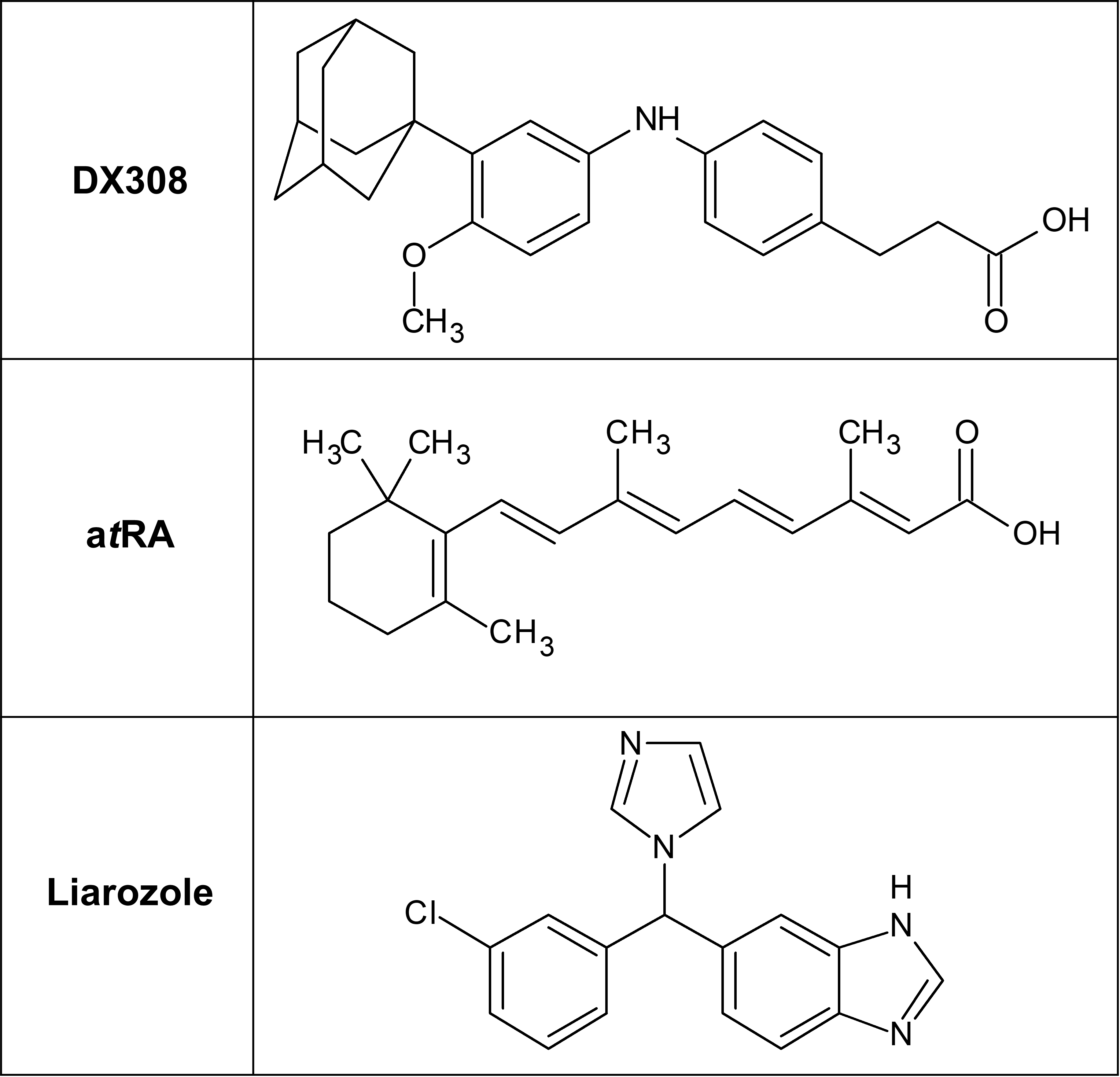


**Figure S1: Chemical structure of DX308, all-*trans*-retinoic acid (a*t*RA), and liarozole.**


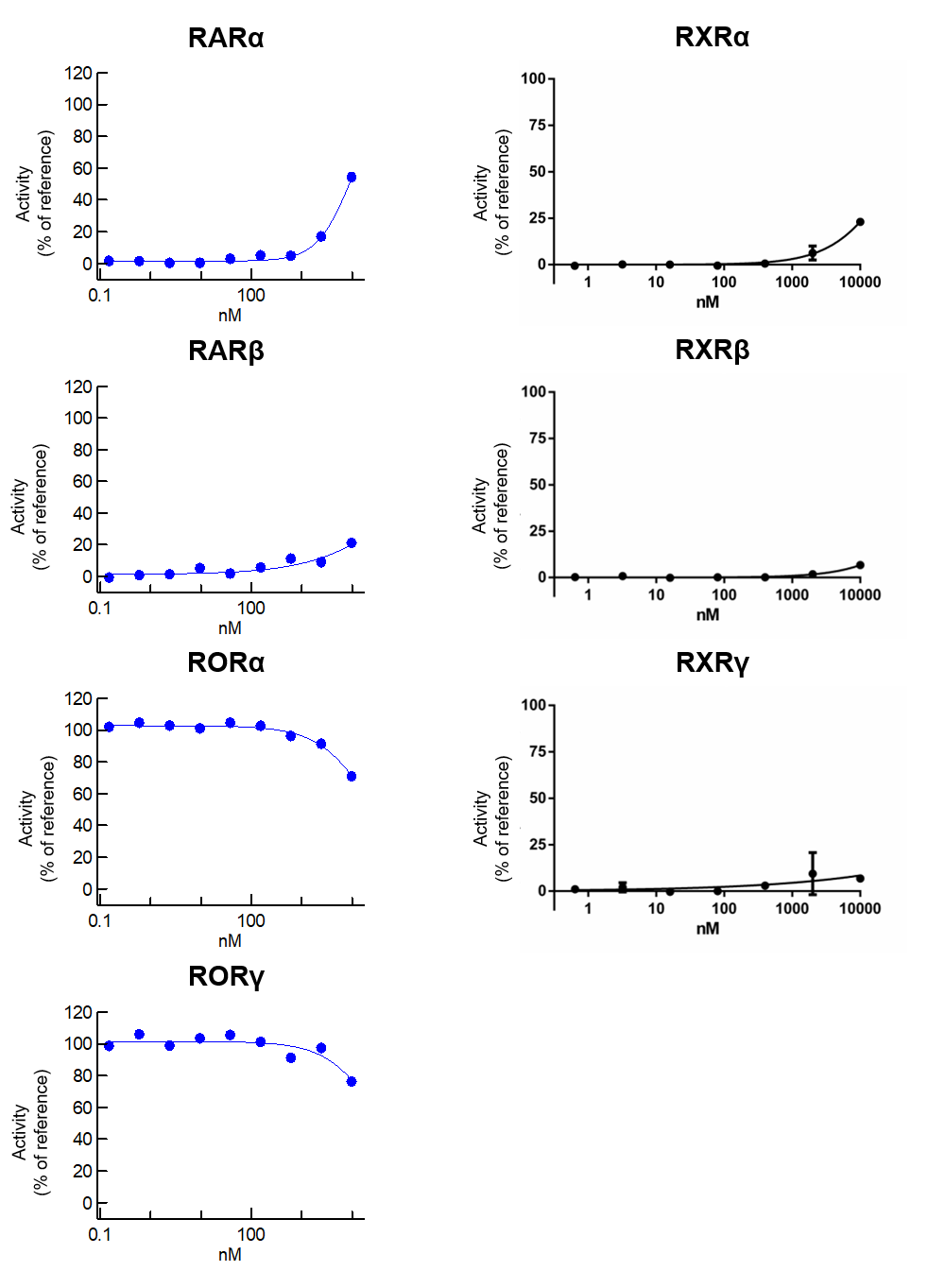


**Figure S2: Logistic regression curves of DX308 activity on nuclear receptors RARα/β, RORα/γ, and RXRα/β/γ by luciferase reporter assay.**

**
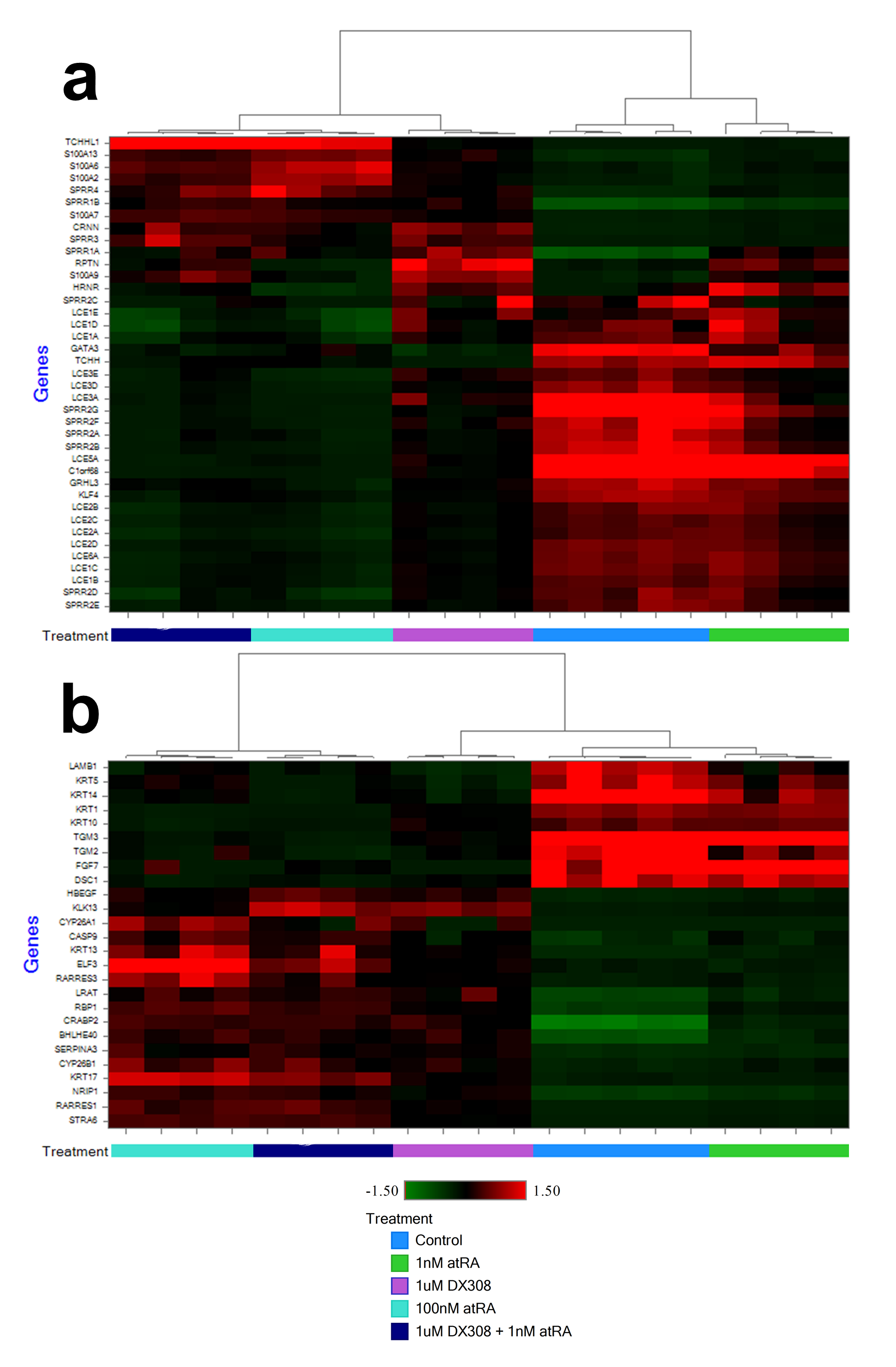
**

**Figure S3: Hierarchical clustering of significantly differentially expressed transcripts in healthy RHE.** **(a)** Epidermal differentiation complex genes or **(b)** genes involved in keratinization. Expression shown in standardized TPM. Significantly differentially expressed transcripts are those with a |fold-change| ≥2 and FDR <0.05. a*t*RA, all-*trans* retinoic acid; FDR, false discovery rate; TPM, transcripts per million.
